# Opening for a Physician

**Published:** 1869-02

**Authors:** 


"OPENING FOR A PHYSICIAN."—We are requested to announce that Drs. Wallace and Sage of Elery, Chautauqua county, N. Y., will discontinue the practice of medicine, and that they both desire that a good physician should locate in that place.

A rebellious case of aphonia, instantly cured by electrical excitation of the inferior laryngeal nerve, has been communicated by Dr. R. Philipeaux to the *Gazette Medicale de Lyon*, 1868, No. 30.

Dr. Moore, late Surgeon-General of the Confederate Army, has been elected Superintendent of the Eastern Lunatic Asylum of Virginia.

